# A quality assessment of orthodontic patient information leaflets

**DOI:** 10.1186/s40510-016-0128-y

**Published:** 2016-05-16

**Authors:** Jadbinder Seehra, Laura Cockerham, Nikolaos Pandis

**Affiliations:** King’s College London, Department of Orthodontics, Dental Institute, Strand, London, WC2R 2LS United Kingdom; Department of Orthodontics, Kings College Hospital NHS Foundation Trust, Bessemer Road, London, SE5 9RS United Kingdom; Department of Orthodontics and Dentofacial Orthopedics, Dental School/Medical Faculty, University of Bern, Freiburgstrasse, Bern, 7 CH-3010 Switzerland

**Keywords:** DISCERN, Orthodontic, Patient, Leaflets

## Abstract

**Background:**

Patient information leaflets (PILs) are often used to reinforce and provide further information relating to treatment choices, risks, and alternatives. An assessment of the quality of commonly used orthodontic patient information leaflets is lacking.

**Methods:**

A cross-sectional assessment of patient information leaflets from two international orthodontic societies was undertaken. The quality of each leaflet was assessed using the DISCERN instrument. The readability of each leaflet was assessed using the Flesch Reading Ease instrument, Flesch-Kincaid Grade Level and Simple Measure of Gobbledygook (SMOG) index. Descriptive statistics followed by univariate analysis was conducted.

**Results:**

Thirty-six patient information leaflets were identified. Reporting of DISCERN instrument items relating to aims, description of sources, details of additional sources, consequences of no treatment, possible treatment options, and support for a shared decision process was of low quality. The overall quality score for the total sample was 44. The median Flesch Reading Ease, Flesch-Kincaid Grade Level, and SMOG index scores were 70 (interquartile range (IQR) 53.3–73.9), 7.2 (IQR 6–9.7), and 7.3 (IQR 6.7–9.1), respectively. There was a significant difference between the quality (−8.00, 95 % CI: −14.62, −1.38, *p* < 0.001), Flesch Reading Ease (−22.30, 95 % CI: −26.77, 17.83, *p* < 0.001) and the Flesch-Kincaid Grade Level (3.80, 95 % CI: 2.74, 4.86, *p* < 0.001) scores between the two societies’ PILs.

**Conclusions:**

In relation to the DISCERN instrument, the quality of orthodontic PILs is deemed of moderate quality. There is a significant difference between the quality scores and the readability of PILs from different societies.

## Background

Supplemental written information is utilised during health-care consultations and can affect health outcomes [1]. It is recommended that verbal information should be supported by written and/or visual information [2]. The importance of this is highlighted by the finding that patient’s retention of information conveyed during health-care consultations can be limited and variable [3, 4]. Patient information leaflets (PILs) are frequently used during orthodontic consultations. The benefits of PILs include increased retention of clinical information [4], improved patient communication and satisfaction [5], and potential reduction in patient anxiety [6]. PILs may also contribute to the informed consent process [7]. The importance of informing of patients of the material risks involved in treatment and reasonable alternatives has been recently highlighted [8].

Despite their perceived benefits, PILs are underutilised and poorly written [1]. It is imperative that health professionals are aware of the accuracy, quality, and limitations of PILs that they use [9]. PILs should also be based on the most current scientific evidence [10]. The DISCERN instrument is a reliable and valid tool developed to allow both providers of health care and patients to assess the quality of written information relating to treatment choices [10].

Effective PILs are those which are accessible, easily understood by a wide-ranging readership and designed at the appropriate reading age [1]. The Flesch Reading Ease [11] and the Flesch-Kincaid Grade Level [12] are valid and reproducible methods of assessing both readability and level of comprehension difficulty. A weighted score for both readability and reading age is calculated using the number of words and sentence length of a piece of text. The average reading age of the US and UK populations is the eighth grade (13–14 years old) and patient information should be aimed approximately at grade 6 and not exceed a reading age of 12 [1, 13].

An assessment of the quality of orthodontic PILs has not been previously undertaken. The primary aim of this study was to evaluate the quality of orthodontic PILs with reference to the DISCERN instrument. A secondary aim was to assess the readability of each patient information leaflet. The null hypothesis of this study was that there is no difference in the quality as assessed using the DISCERN instrument and readability of orthodontic PILs.

## Methods

The most recent electronic versions of orthodontic PILs were identified from two international orthodontic societies, British Orthodontic Society (BOS) and American Association of Orthodontists (AAO). Two investigators (LC and LB) screened potentially relevant PILs independently, and any disagreements were resolved by discussion with a third author (JS) to reach a consensus. The investigators (LC and LB) were calibrated by assessing the reporting of five PILs together by referring directly to the DISCERN instrument [10] and the associated explanation. Inter-examiner reliability was assessed (mean difference 0.163).

The DISCERN instrument comprises of a 16-item questionnaire, divided into three domains: reliability (items 1–8), specific information related to treatment choices (items 9–15), and an overall rating of the quality (item 16) of the publication. Each item is rated on a five-point scale from 1 (low quality with serious or extensive shortcomings) to 5 (high quality with minimal shortcomings). Each item on the DISCERN instrument for individual PILs was scored independently by two authors (LC and LB) producing an overall score for each PIL. Disagreements were resolved by consulting with a third author (JS). Similar to previous research [14], a summative score from questions 1 to 15 was obtained giving a total score of ranging between 15 and 75, where a score of 15 was deemed very poor quality and 75 was very high quality. All data were collected using a pre-specified data collection form. One author (LC) assessed the readability of the PILs. The entire text from the pdf versions of each PIL was copied into a Word (Microsoft office, Version 15.13.1) document. These Word documents were cross-checked against the pdf versions to ensure completeness. The text was then imported into an online tool (www.readability-score.com). The following assessment methods/formulae were used to assess readability and reading grade level: Flesch Reading Ease [11], Flesch-Kincaid Grade Level [12] and Simple Measure of Gobbledygook (SMOG) index [15]. The Flesch readability tools utilise the sentence length (number of words per sentence) and the number of syllables per word in specific equations with different weighting factors to calculate the reading ease and grade level respectively. Similarly, in the SMOG index, 30 sentences (ten from the beginning, middle and near the end) from the identified reading material are selected. Within these sentences every word with three of more syllables is identified. The total number of words in the material is also calculated. Using these variables, either the SMOG conversion table or equation is used to calculate the reading level. The Flesch Reading Ease score has a range of 0 to 100, where 0 represents a very difficult passage to read and 100 a very simple one [16]. The overall readability of a PIL based on the Flesch Reading Ease score was classified as difficult (score below 50), fairly difficult (score above 60) and comfortable reading text (score above 80) [17].

### Statistical analysis

Descriptive statistics for individual reporting items and each PIL were calculated. The calculated score was non-normally distributed. Univariate median regression was implemented to identify characteristics associated with mean score. A two-tailed *p* value of 0.05 was considered statistically significant and analyses were performed using STATA 12.1 (Stata Corporation, College Station, Texas, USA).

## Results

A total of 36 (21 BOS and 15 AAO) orthodontic patient leaflets were identified and included in this study. The ratings of individual DISCERN items are shown in Table 1. For item 16 (overall quality rating), the most frequent score achieved was 3 (50.0 %) followed by 4 (44.4 %). No patient information leaflet achieved a rating of 5. The summative quality score for each orthodontic PIL is shown in Table 2. The average overall quality score for the total sample was 44. The median score for BOS and AAO PILs was 47 (interquartile range (IQR) 40–50) and 39 (IQR 36–45), respectively. There was a significant difference in the quality scores between BOS and AAO PILs, with lower scores achieved by AAO PILs compared to BOS PILs (−8.00, 95 % CI: −14.62, −1.38, *p* < 0.001) (Table 3).

**Table 1:** The ratings of individual DISCERN items (*N* = 36)

	Rating of 1 and 2 (low quality) *N* (%)	Rating of 3 (moderate quality) *N* (%)	Rating of 4 and 5 (high quality) *N* (%)
DISCERN item			
1) Are the aims clear?	17 (47.2 %)	14 (38.9 %)	5 (13.9 %)
2) Does the leaflet achieve its aims?^a^	0 (0.0 %)	0 (0.0 %)	33 (91.2 %)
3) Is it relevant?	0 (0.0 %)	2 (5.6 %)	34 (94.4 %)
4) Is it clear what sources of information were used to compile the publication (other than the author or producer)?	36 (100 %)	0 (0.0 %)	0 (0.0 %)
5) Is it clear when the information used or reported in the publication was produced?	6 (16.7 %)	30 (83.3 %)	0 (0.0%)
6) Is it balanced and unbiased?	1 (2.8 %)	15 (41.7 %)	20 (55.5 %)
7) Does it provide details of additional sources of support and information?	25 (69.4 %)	6 (16.7 %)	5 (13.9 %)
8) Does it refer to areas of uncertainty?	2 (5.5 %)	5 (13.9 %)	29 (80.6 %)
9) Does it describe how each treatment works?	5 (13.9 %)	9 (25.0 %)	22 (61.1 %)
10) Does it describe the benefits of each treatment?	2 (5.6 %)	16 (44.4 %)	18 (50.0 %)
11) Does it describe the risks of each treatment?	12 (33.3 %)	8 (22.2 %)	16 (44.5 %)
12) Does it describe what would happen if no treatment is used?	18 (50.0 %)	10 (27.8 %)	8 (22.2 %)
13) Does it describe how the treatment choices would affect overall quality of life?	7 (19.4 %)	16 (44.4 %)	13 (36.1 %)
14) Is it clear that there may be more than one possible treatment choice?	14 (38.8 %)	11 (30.6 %)	11 (30.6 %)
15) Does it provide support for shared decision-making?	22 (61.1 %)	10 (27.8 %)	4 (11.1 %)

^a^Not applicable for three leaflets

**Table 2:** The summative DISCERN score for each PIL

Patient information leaflet title	Source	Summative score
Adult orthodontics	BOS	41
Caring for your child’s teeth	BOS	50
Dummy and thumb sucking habits	BOS	43
Your first visit to the orthodontist	BOS	42
Fixed appliances	BOS	39
Teeth and brace-friendly food and drink	BOS	56
Functional appliances	BOS	40
Headgear	BOS	37
Hypodontia	BOS	50
Impacted canines	BOS	53
Interproximal reduction	BOS	53
How to keep your teeth and gums healthy	BOS	54
Orthognathic surgery	BOS	48
Orthodontic treatment	BOS	47
Protraction headgear	BOS	40
Removable appliances	BOS	37
Retainers	BOS	53
Orthodontic treatment—what are the risks?	BOS	48
Rapid maxillary expansion (RME)	BOS	47
Tooth transplants	BOS	50
Orthodontic mini-screws	BOS	39
The right time for orthodontic check up	AAO	39
What is an orthodontist	AAO	37
Adult orthodontics	AAO	36
Your child’s first orthodontic check up: no later than age 7	AAO	35
All about orthodontics	AAO	34
Frenectomies, friberotomies and gingivoplasties	AAO	44
Orthodontic headgear	AAO	39
Keeping your smile beautiful	AAO	46
Palatal expansion	AAO	50
Show your smile	AAO	37
Temporary anchorage devices (TAD)	AAO	45
Keeping your teeth clean	AAO	44
Prevent accidents	AAO	47
Elastics	AAO	36
Interproximal reduction	AAO	45

**Table 3:** Univariate median regression-derived coefficients (*β*) and 95 % confidence intervals (CI) for quality scores as dependent variables for the 36 PILs

Predicator variables		Univariate analysis		
Variable	Category	*β*	95 % CI	*p* value
DISCERN scores	BOS	Baseline (reference)		
	AAO	−8.00	−14.62, −1.38	<0.001

For the total sample, the median Flesch Reading Ease, Flesch-Kincaid Grade Level, and SMOG index scores were 70 (IQR 53.3–73.9), 7.2 (IQR 6–9.7), and 7.3 (IQR 6.7–9.1) respectively. The median Flesch Reading Ease, Flesch-Kincaid Grade Level, and SMOG index scores for both BOS and AAO PILs are shown in Table 4. There was a significant difference in the Flesch Reading Ease scores between BOS and AAO PILs, with lower scores achieved by AAO PILs compared to BOS PILs (−22.30, 95 % CI: −26.77, −17.83, *p* < 0.001). Both the Flesch-Kincaid Grade Level (3.80, 95 % CI: 2.74, 4.86, *p* < 0.001) and SMOG index (2.60, 95 % CI: 1.82, 3.38, *p* < 0.001) scores for AAO PILs were significantly higher than BOS PILs (Table 5).

**Table 4:** Median readability scores for BOS and AAO PILs

Leaflet source	Readability tool	Median	IQR
BOS (*N* = 21)	Flesch Reading Ease	73.1	71.5–76
	Flesch-Kincaid Grade Level	6.3	5.9–6.9
	SMOG index	6.7	6.4–7.2
AAO (*N* = 15)	Flesch Reading Ease	50.8	46–56.1
	Flesch-Kincaid Grade Level	10.1	9.1–11.5
	SMOG index	9.3	8.9–10.7

**Table 5:** Univariate median regression-derived coefficients (*β*) and 95 % confidence intervals (CI) for readability scores as dependent variables for the 36 PILs

Predicator variables		Univariate analysis		
Variable	Category	*β*	95 % CI	*p* value
Flesch Reading Ease	BOS	Baseline (reference)		
	AAO	−22.30	−26.77, −17.83	<0.001
Flesch-Kincaid Grade Level	BOS	Baseline (reference)		
	AAO	3.80	2.74, 4.86	<0.001
SMOG index	BOS	Baseline (reference)		
	AAO	2.60	1.82, 3.38	<0.001

## Discussion

It is paramount that written information should be focused at those who would most benefit from them as part of their planned health care and clinicians who utilise this information are confident of its quality [1]. The DISCERN instrument has been developed to aid the production of high-quality evidence-based consumer information by setting standards and providing a reference point for authors [10]. The primary aim of this study was to assess the quality of orthodontic PILs with reference to the DISCERN instrument. When assessed in relation to the DISCERN instrument the quality of PILs used in both medical and dental specialties has been reported to be sub-optimal [14, 16, 17]. The secondary aim was to assess both readability and reading age of PILs. Both variables have been investigated previously in both medical and dental literature with a large variance reported [13, 14, 18–20].

Based on the overall summative score and most frequent score for item 16 (overall quality rating) of the DISCERN instrument, the orthodontic PILs included in this study were deemed to be of moderate quality. The mean overall quality score for the total sample was 44 which is comparable to mean quality score of medical PILs of 35.2 reported by Rees et al. [14]. Similarly, no patient information leaflet achieved a rating of 5 for overall quality (item 16), which is consistent with previous studies [14]. PILs produced by the BOS were of higher quality compared to AAO. Our study has also highlighted deficiencies in the quality of PILs in relation to particular items of the DISCERN instrument. Items assessed as low quality included description of aims (47.2 %), description of sources (100 %), details of additional sources (69.4 %), consequences of no treatment (50 %), possible treatment options (38.8 %), and support for a shared decision process (61.1 %). Similar findings have been reported in an assessment of dental PILs. Lewis and Newton [16] reported deficiencies in reporting aims, reference to sources of information or date of production, risks of treatment, effect of choosing not to have treatment, effect of treatment on overall quality of life, and support for shared decision-making. As part of the informed consent process, patients should now be informed of potential material risks of treatment [8]. PILs have been reported to contribute to the informed consent process [7]. However, in relation to the DISCERN instrument, orthodontic PILs appear to be lacking sufficient information relating to treatment options, consequences of no treatment and support for shared decision-making which may affect the validity of the consenting process.

The readability of written material should not exceed the reading age of 12 years old and for US and UK populations ideally be aimed at eighth grade (13–14 years old) and grades 5–6 (10–11 years old), respectively [1, 13]. Previous investigations of dental practice leaflets readability have found leaflets to be difficult to read with a mean Flesch Reading Ease level ranging between 55.2 (SD 12.5) and 72.19 (SD 4.75) [16, 18]. Similarly, UK hospital patient information leaflets have been assessed to have a mean Flesch-Kincaid reading grade of 7.8 and deemed to exceed the comprehension of readers [13].

In this study, the median Flesch Reading Ease, Flesch-Kincaid Grade Level, and SMOG index scores were 70 (IQR 53.3–73.9), 7.2 (IQR 6–9.7), and 7.3 (IQR 6.7–9.1) respectively. In relation to the UK population this suggests the PILs included in this study are written at not the appropriate reading age and are fairly difficult to read. Differences between the readability of PILs from the two societies were also detected. In comparison to the BOS PILs, the AAO PILs achieved lower Flesch Reading Ease scores (−22.30, 95 % CI: −26.77, 17.83, *p* < 0.001) and higher Flesch-Kincaid Grade Level (3.80, 95 % CI: 2.74, 4.86, *p* < 0.001) and SMOG index (2.60, 95 % CI: 1.82, 3.38, *p* < 0.001) scores. These findings suggest AAO PILs are fairly difficult to read and aimed at individuals with a higher reading age compared to BOS PILs and the recommended reading age level (Tables 3 and 4). For the total sample there was no apparent difference between the Flesch-Kincaid Grade Level and SMOG index scores. When the BOS PILs and AAO PILs were compared a slightly lower SMOG score was detected. Previous readability assessments of dental practice leaflets have reported the reverse finding and attributed to this to the higher level of comprehension that is required to validate the SMOG test resulting in a higher score [16]. This apparent disparity confirms that the results of readability formulae only provide an estimate of reading level and should be used as a guide [16].

The results of this study are very similar to those reported in a previous investigation of the readability of orthodontic PILs [20]. In this study, the mean Flesch Reading Ease score for BOS PILs was 70.8 (SD 4.6) and AAO PILs 43.9 (SD 5.2). The mean Flesch-Kincaid reading grade scores for BOS PILs was 6.6 (SD 0.7) and AAO PILs 10.9 (SD 0.7). This finding may be unsurprising as the majority of PILs included in both studies are the same. However, the results appear to further support the long-term reliability and validity of the Flesch reading tools. The current study was undertaken 10 years after the study conducted by Harwood and Harrison [20] and it appears that improvements to enhance the readability of PILs have not been implemented.

All patient information leaflets from two orthodontic societies were only included in this study which may result in an element of selection bias. The decision to include leaflets from these two societies was based on accessibility. Future studies may include leaflets from additional societies and practices hence presenting a wide-ranging assessment of the quality and readability of orthodontic patient information leaflets. The reliability of readability tools has been previously reported [10]. A disadvantage of readability tools is that the majority are usually based on sentence length, syllable count, or vocabulary indexes. A potential bias could be introduced into the findings as a short word could score well using a measure of readability but is not a word that is widely used or understood [1]. To account for this, an evaluation of both readability and comprehension is recommended [1]. In addition, the qualitative score from a lay assessor may also be considered in the assessment of the quality of patient information leaflets. The translation of complex dental/medical terminology into lay terms may not be fully achievable without changing the meaning of the words. A certain degree of health-care terminology may have to be maintained in PILs hence this may result in variation of readability scores when assessed.

## Conclusions

In relation to the DISCERN instrument, the findings of this study have highlighted shortcomings in the quality of orthodontic patient information leaflets. Overall, the quality of patient information leaflets was deemed of moderate quality. However, patient information leaflets produced by the British Orthodontic Society were assessed as higher quality, more readable and designed at the appropriate reading age for the UK population. When using orthodontic patient information leaflets, clinicians may wish to consider providing additional/supplemental information relating to information on consequences of no treatment, possible treatment options and support for a shared decision process.

## References

[1] Kenny T, Wilson RG, Purves IN, Clark J, Newton LD, Newton DP, Moseley DV (1998). A PIL for every ill? Patient information leaflets (PILs): a review of past, present and future use. Fam Pract.

[2] Thomson AM, Cunningham SJ, Hunt NP (2001). A comparison of information retention at an initial orthodontic consultation. Eur J Orthod.

[3] Kitching JB (1990). Patient information leaflets—the state of the art. J R Soc Med.

[4] Gauld VA (1981). Written advice: compliance and recall. J R Coll Gen Pract.

[5] Ley P (1982). Satisfaction, compliance and communication. Br J Clin Psychol.

[6] Jackson C, Lindsay S (1995). Reducing anxiety in new dental patients by means of leaflets. Br Dent J.

[7] Brown H, Ramchandani M, Gillow JT, Tsaloumas MD (2004). Are patient information leaflets contributing to informed consent for cataract surgery?. J Med Ethics.

[8] D’Cruz L, Kaney H (2015). Consent—a new era begins. Br Dent J.

[9] Fitzmaurice DA, Adams JL (2000). A systematic review of patient information leaflets for hypertension. J Hum Hypertens.

[10] Charnock D, Shepperd S, Needham G, Gann R (1999). DISCERN: an instrument for judging the quality of written consumer health information on treatment choices. J Epidemiol Community Health.

[11] Flesch R (1948). A new readability yardstick. J Appl Psychol.

[12] Kincaid JP, Fishburne RP, Rogers RL, Chissom BS (1975). Derivation of new readability formulas (automated readability index, fog count and Flesch Reading Ease formula) for navy enlisted personnel. Research Branch Report.

[13] Williamson JM, Martin AG (2010). Analysis of patient information leaflets provided by a district general hospital by the Flesch and Flesch-Kincaid method. Int J Clin Pract.

[14] Rees CE, Ford JE, Sheard CE (2003). Patient information leaflets for prostate cancer: which leaflets should healthcare professionals recommend?. Patient Educ Couns.

[15] McLaughlin HG (1969). SMOG grading: a new readability formula. J Read.

[16] Lewis MA, Newton JT (2006). An evaluation of the quality of commercially produced patient information leaflets. Br Dent J.

[17] Demir F, Ozsaker E, Ilce AO (2008). The quality and suitability of written educational materials for patients. J Clin Nurs.

[18] Newton JT (1995). The readability and utility of general dental practice patient information leaflets: an evaluation. Br Dent J.

[19] Wong SS, Bekker HL, Thornton JG, Gbolade BA (2003). Choices about abortion method: assessing the quality of patient information leaflets in England and Wales. BJOG.

[20] Harwood A, Harrison JE (2004). How readable are orthodontic patient information leaflets?. J Orthod.

